# The correlation between non-arteritic anterior ischemic optic neuropathy and cerebral infarction

**DOI:** 10.1515/tnsci-2022-0281

**Published:** 2023-03-18

**Authors:** Xiaochun Li, Xiaolu Cao, Fenglou Ma, Peipei Jia, Fuyin Wang, Xiaoguang Cao

**Affiliations:** Department of Ophthalmology, Peking University International Hospital, Beijing, China; Center of Optometry, Department of Ophthalmology, Peking University People’s Hospital, Eye Diseases and Optometry Institute, Beijing Key Laboratory of Diagnosis and Therapy of Retinal and Choroid Diseases, College of Optometry, Peking University Health Science Center, 11# Xizhimen South Street, Xicheng District, Beijing 100044, China; Hebei Ophthalmology Key Lab, Hebei Eye Hospital, No. 399, Quanbei East Street, Xingtai, 054000, Hebei, China; Department of Pharmacy, Hebei Eye Hospital, No. 399, Quanbei East Street, Xingtai, 054000, Hebei, China; Department of Radiology, Hebei Eye Hospital, No. 399, Quanbei East Street, Xingtai, 054000, Hebei, China

**Keywords:** non-arteritic anterior ischemic optic neuropathy, cerebral infarction, hypertension, correlation, brain damage

## Abstract

**Background:**

The aim of this study was to explore the correlation between non-arteritic anterior ischemic optic neuropathy (NAION) and cerebral infarction (CI). Moreover, the ocular and systemic parameters are also compared between NAION patients with or without CI.

**Methods:**

Retrospective analysis is performed for NAION patients and the controls. The controls were collected at the eye outpatient with cranial computed tomography (CT), and data of blood triglyceride, cholesterol, low-density lipoprotein, high-density lipoprotein, and apolipoprotein B were drawn. The diagnosed NAION patients with cranial CT are included, and data of clinical history and routine clinical examination were drawn from the medical record. Visual acuity, intraocular pressure (IOP), visual field, and visual evoked potential were also drawn.

**Results:**

Eighty-two unilateral and 6 bilateral patients, totally 94 eyes for 88 NAION patients and 69 controls are included. NAION and control patients have matched age, gender, and weight. There is no difference in triglyceride, cholesterol, low-density lipoprotein, high-density lipoprotein, and apolipoprotein B between these two groups. NAION patients (43.18%, 38/88) have a higher ratio of CI than the controls (14.49%, 10/69) (*p* = 0.000). For NAION, the odds ratio (OR) of CI is 2.691 (*p* = 0.011). Body mass index, height, and IOP show no significant difference between NAION patients with or without CI. NAION patients with CI have a significant higher ratio of hypertension than those without CI, and the OR of HBP is 2.623 (*p* = 0.008).

**Conclusions:**

The correlation between NAION and CI is possible as NAION patients have a significant higher ratio with CI. In NAION patients, hypertension is a risk factor for those with CI.

Ischemic optic neuropathy (ION) is one of the major causes for the blindness, as the two broad varieties namely posterior and anterior ischemic optic neuropathy (AION). AION refers to ischemia of small branches deriving from the posterior ciliary vessels supplying the anterior and ethmoid regions of optic disc, resulting in local infarctions of it. It is a group of syndromes characterized by sudden vision loss, optic disc edema, and characteristic visual field (VF) defects (fan-shaped defects connected with physiological blind spots). The disease usually occurs in the middle-aged or elderly, and often in both eyes successively, with an interval of weeks, months, or years. It is generally caused by hypertension, arteriosclerosis, diabetes, increased blood viscosity, severe anemia, low blood pressure, increased intraocular pressure (IOP), and other factors. As the arteritic variety of AION (AAION) is usually due to giant cell arteritis, non-arteritic anterior ischemic optic neuropathy (NAION) is very common optic neuropathy in adults [1,2].

The etiology of NAION is not definitively known [3,4]. Despite extensive previous studies, the most reasonable thought indicates that NAION is caused by an infarction in the region of optic nerve head [5]. As the cerebral infarction (CI) is also due to the vessel abnormality, there might have a possible correlation between NAION and CI.

Our study is trying to explore the possible correlation between NAION and CI. Moreover, the ocular and systemic parameters are also compared between NAION patients with or without CI.

## 1 Methods

### 1.1 Recruitment of patients, and inclusion and exclusion criteria

This retrospective study is conducted in Hebei Eye Hospital, Hebei, China.

All included patients are retrospectively enrolled in this study, January 2020 to June 2022. For NAION patients, the inclusion criterion includes: diagnosed as NAION unilateral or bilateral and documented results of cranial computed tomography (CT). For the control, the patients visiting the eye outpatient due to other reasons, who have documented results of cranial CT, are included. Exclusive criterion is without documented results of cranial CT.

### 1.2 Clinical collections

Data of clinical history and routine clinical examination were drawn from the medical record. For the controls, data of cranial CT, results of blood triglyceride, cholesterol, low-density lipoprotein, high-density lipoprotein, and apolipoprotein B were drawn. For NAION patients, beside those, visual acuity (VA), IOP, VF, and visual evoked potential (VEP) were also drawn.

### 1.3 VF, VEP, and CT measurement

VF was tested with Octopus 900 perimeter (Haag-Streit AG, formerly Interzeag AG, Schlieren, Switzerland). The program of White-on-White TOP strategy with 4/1,000 asb III 100 ms and Octopus G Standard distribution of points was performed for those included patients. The acceptable criterion of reproducible test is both of false-positive and false-negative response rates <15%. Mean deviation (MD), mean sensitivity (MS), and square root of loss variance (sLV) were drawn from the reports.

According to ISCEV (International Society for Clinical Electrophysiology of Vision) standard, RETI-Port/Scan 21 multifocal visual electrophysiology examination system (Roland Consult, Stasche&Finger GmbH, Germany) was used for VEP measurement.

CT was performed at the Department of Radiology using SOMATOM Definition Edge (Siemens AG, Germany).

### 1.4 Statistical analysis

The Kolmogorov–Smirnov test was used to verify the normality of data distribution. For quantitative comparisons between groups, we used the Student’s *t*-test for independent samples in parametric variables and independent Mann–Whitney *U* test for the non-parametric variables. Pearson correlation coefficients were calculated to assess the relation between variables. Binary logistic regression is calculated to assess the influence. Statistical analyses were performed using SPSS statistical software for Windows (version 20.0, IBM-SPSS, Chicago, IL, USA). The level of statistical significance was set at *p* < 0.05. Power analysis was calculated at the website “http://powerandsamplesize.com/”.


**Ethical approval:** The research related to human use has been complied with all the relevant national regulations, institutional policies, and in accordance the tenets of the Helsinki Declaration and has been approved by the authors’ institutional review board or equivalent committee and has been approved by the institutional review board of Hebei Eye Hospital.
**Informed consent**: Informed consent has been obtained from all individuals included in this study.

## 2 Results

The included patients were set as two groups, NAION and control groups. The NAION group had 82 unilateral and 6 bilateral patients, totally 94 eyes of 88 patients. The control group had 69 patients. As the data shown in Table 1, the mean ages of the NAION and control groups were 57.22 ± 9.96 and 54.91 ± 13.32 years (*p* = 0.268). Female ratios of the NAION and control groups are no significant difference (female/male, 43/45 and 42/27, *p* = 0.134). The weights of NAION and control groups are no significant difference (*t* = 0.982, *p* = 0.328).

**Table 1:** The characteristics of non-arteritic anterior ischemic optic neuropathy (NAION) and control patients

	NAION			Control		
	Total	With CI	Without CI	Total	With CI	Without CI
Age (years)	57.22 ± 9.96	59.61 ± 9.87	55.16 ± 9.68	54.91 ± 13.32	66.90 ± 14.14	52.81 ± 12.26
Weight (kg)	69.06 ± 12.26	70.41 ± 11.55*	68.13 ± 12.90	71.15 ± 13.78	61.30 ± 14.46*	73.05 ± 13.09
Triglyceride (mmol/L)	1.67 ± 1.03	1.61 ± 1.18	1.72 ± 0.90	1.50 ± 0.74	1.35 ± 0.44	1.56 ± 0.79
Cholesterol (mmol/L)	4.72 ± 0.94	4.63 ± 0.95	4.80 ± 0.94	4.83 ± 1.25	5.20 ± 2.42	4.76 ± 0.87
LDL (mmol/L)	3.18 ± 0.87	3.13 ± 0.87	3.22 ± 0.87	3.33 ± 0.97	3.19 ± 1.18	3.38 ± 0.95
HDL (mmol/L)	1.23 ± 0.27	1.23 ± 0.23	1.23 ± 0.27	1.23 ± 0.30	1.21 ± 0.46	1.19 ± 0.28
Apolipoprotein B (g/L)	0.86 ± 0.20	0.84 ± 0.20	0.88 ± 0.21	0.91 ± 0.26	0.90 ± 0.33	0.91 ± 0.25

CI, cerebral infarction; LDL, low-density lipoprotein; HDL, high-density lipoprotein. ^*^ *p* < 0.05.

In the results of blood biochemical test, the differences between the NAION and control groups of triglyceride, cholesterol, low-density lipoprotein, high-density lipoprotein, and apolipoprotein B are not significant. Table 1 shows the age, weight, triglyceride, cholesterol, low-density lipoprotein, high-density lipoprotein, and apolipoprotein B.

**Figure 1:**
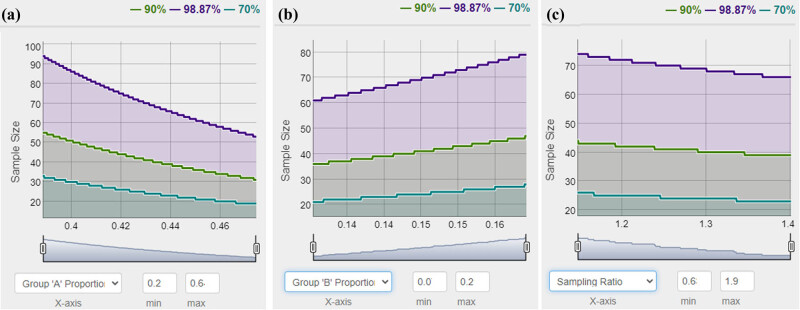
Power analysis for the sample size on the groups of NAION and control with/without CI. (a) Group of NAION; (b) group of control; and (c) sampling ratio.

As the results of cranial computed tomography (CT), CI or ischemia is detected in 38 patients of the NAION group and 10 patients of the control group. The prevalence of CI is 43.18% (38/88) for NAION patients and 14.49% (10/69) for the controls. NAION patients have a higher ratio of CI than the controls (*p* = 0.000). Based on the samples and *α* = 0.05 (type I error rate), the power (1 − *β*) = 0.9887 (98.87%) as Figure 1. The correlation between NAION and CI is explored by using the multivariate logistic regression model. For NAION, the odds ratio (OR) of CI is 2.691 (*p* = 0.011).

Table 2 shows the differences between the NAION patients with or without CI, and Table 3 shows the systemic disorders for the NAION patients with or without CI. NAION patients with CI have a higher HBP percent (*p* = 0.004). For NAION, the OR of HBP is 2.623 (*p* = 0.008).

**Table 2:** The ocular and systemic information of non-arteritic anterior ischemic optic neuropathy (NAION) patients with or without cerebral infarction (CI)

NAION	without CI	with CI
BMI (kg/cm^2^)	25.80 ± 3.65	25.76 ± 3.39
Height (cm)	163.36 ± 7.29	165.32 ± 8.08
IOP (mmHg)	15.50 ± 2.73	16.03 ± 3.28
MD of visual filed (dB)	17.18 ± 7.65	17.25 ± 6.84
MS of visual filed (dB)	10.66 ± 7.77	10.28 ± 6.92
sLV of visual filed (dB)	6.41 ± 2.93	8.38 ± 10.55
CRP (mg/L)	3.30 ± 13.71	1.82 ± 3.45
Whole blood viscosity low cut (mPa s)*	11.80 ± 4.35	9.21 ± 2.71
Whole blood viscosity mid cut (mPa s)	6.27 ± 1.52	5.65 ± 1.35
Whole blood viscosity high cut (mPa s)	4.93 ± 1.06	4.67 ± 0.82
Plasma viscosity (mPa s)	1.45 ± 0.19	1.35 ± 0.21
ESR (mm/h)	12.72 ± 10.61	14.00 ± 11.33
Whole blood reduced viscosity low cut (mPa s)*	24.51 ± 8.33	18.25 ± 6.45
Whole blood reduced viscosity mid cut (mPa s)	11.45 ± 2.73	10.04 ± 2.97
Whole blood reduced viscosity high cut (mPa s)	8.39 ± 2.07	7.88 ± 1.68
Hematocrit	37.77 ± 12.50	42.22 ± 3.75
Erythrocyte aggregation index EAI *	2.40 ± 0.63	1.94 ± 0.26
Latent period of p100 (ms)	118.60 ± 20.95	108.82 ± 17.34
Latent period of N2 (ms)	71.41 ± 21.92	72.13 ± 23.44
Latent period of P2 (ms)	111.58 ± 22.05	109.37 ± 13.84
Amplitude of N75-P100 (μV)	6.79 ± 4.30	7.87 ± 5.30
Amplitude of P100-N135 (μV)	6.72 ± 5.14	8.24 ± 6.23
Amplitude of N2-P2 (μV)	7.93 ± 3.68	9.99 ± 4.89
VA at diagnosis (decimal)	0.22 ± 0.27	0.17 ± 0.25
VA after treatment (decimal)	0.37 ± 0.33	0.34 ± 0.31
Changed VA (decimal)	0.15 ± 0.16	0.17 ± 0.20

NAION, non-anterior ischemic optic neuropathy; BMI, body mass index; IOP, intraocular pressure; CRP, C-reactive protein; ESR, erythrocyte sedimentation rate; VA, visual acuity; CI, cerebral infarction. *p < 0.05.

**Table 3:** The systemic disorders for non-arteritic anterior ischemic optic neuropathy (NAION) patients with or without cerebral infarction (CI)

NAION	Without CI (yes/no, %)	With CI (yes/no, %)
HBP*	20/30, 40.00%	27/11, 71.05%
DM	20/30, 40.00%	12/26, 31.58%
Hyperlipidemia	3/47, 6.00%	2/36, 5.26%
Hypertriglyceridemia	1/49, 2.00%	0/38, 0.00%
Hypercholesterolemia	0/50, 0.00%	0/38, 0.00%
High LDL	0/50, 0.00%	0/38, 0.00%

HBP, hypertension; DM, diabetes mellitus; LDL, low-density lipoprotein. *p < 0.05.

## 3 Discussions

As a rapid onset ocular disorder, NAION induces serious damage of visual function and poor visual prognosis. In recent years, its incidence age has a younger trend, and that prevalence has increased. The occurrence of this disease has certain age, gender, and ethnic characteristics [6]. NAION tends to occur in middle-aged or elderly people over 45 years old, and the prevalence of man is higher than that of woman. In the United States, the prevalence of people over 50 years old is 23–102 per million [7]. According to the previous studies, the prevalence in Chinese adult is 1:16,000 [8]. Hayreh reported that the incident age of NAION is mostly 60 ± 15 years old, and 45–65 years old can account for 50% [9]. Moreover, some studies reported that the average age of patients in China was 51 years old, with males accounting for 68.80%. The age of onset was lower than that reported previously. In addition, Caucasian patients account for 95% of NAION patients in the United States [10], which shows that there are differences in the incidence between races. NAION patients included in our study are 57.22 ± 9.96 years old, their age is consistent with those previous studies, might be older than some previous results in China. In our study, the NAION patients have 82 unilateral and 6 bilateral, which is consistent with previous studies [11].

At present, the pathogenesis of this disease is not clear, and there are various hypotheses. It can be clear that NAION is caused by the short interruption of optic papilla circulation due to the hypoperfusion and ischemia [5]. Some studies observed the dynamic changes of capillary network on the surface of optic disc using optical coherence tomographic angiography (OCTA) [12,13,14]. The results show that the signal of main retinal vessels is reduced, and the superficial capillaries around the optic papilla are dilated. Thus, the pathogenesis of NAION is related to the blood perfusion at the optic papilla. CI, especially lacunar cerebral infarction (LCI), has a similar pathological mechanism, the disorders of small vessels [15,16,17,18]. LCI refers to the pathological changes of small perforating arteries in the deep part of brain, which eventually form thrombus or microembolism, leading to vascular occlusion and lacunar softening lesions. The diameter of those arteries is generally 2–15 mm, and the maximum is not more than 20 mm. Epidemiological studies show that the incidence rate of LCI in China is 78/100,000, and the proportion of LCI in all CI and ischemic infarction is 27.6 and 36.9% respectively.

A recent study with 10-year follow-up reported 12,150 first-ever LCIs were diagnosed in 489,597 Chinese adults [19]. An epidemiologic study in Beijing reported the prevalence of cerebrovascular disease was 21.4% in aged 60 years old and above Chinese. HBP was the most common related factor in it. Another study in Beijing reported 16% silent CI in aged 35 years old and above with MRI diagnosed. Aging, HBP, DM, and smoking are the independent risk factors, and a previous stroke was reported by 235 individuals (7.33%) in the Beijing Eye Study [20]. The controls in our study are age and gender matched with those included NAION patients, have a higher ratio of CI, 14.49% (10/69). This number is different from those previous studies in Beijing. The reason might be that the controls in our study are collected from the ophthalmic outpatient medical records. Nevertheless, NAION patients in our study have a much higher ratio of CI, 43.18% (38/88). It is the first report for the CI prevalence in NAION patients, and also strong evidence that NAION is closely related to CI. The correlation between NAION and CI should be explored deeply. Moreover, ocular signs and symptoms may be a warning sign of cardiovascular or cerebrovascular events, namely stroke [21]. Previous studies corroborated our finding from another perspective as several studies had shown that following NAION, the increased risks for cardiovascular disease and cerebral vascular accident could be expected. Moreover, taking aspirin might eliminate this expectation [22].

NAION patients with CI show a higher ratio of HBP than those without CI in our study. HBP has been reported as a risk factor not only for NAION but also for CI [23,24]. HBP is the direct cause of LCI. The vascular stenosis caused by the sclerosis of fundus arteries, secondary to HBP, also increases the risk of NAION [25]. DM, hyperlipidemia, hypertriglyceridemia, hypercholesterolemia, and high LDL have no difference for NAION patients with or without CI in our study. The reason may be the bias due to the small volume of included patients. At the diagnosis, NAION patients with CI show a lower VA than those without CI, no significance. It might be the result of CI, the damage of central nervous system, and also a forecast of worse prognosis.

There are some limitations to our study. First, the smaller number of participants may introduce some selection bias. Second, the controls were collected from the eye outpatient. It does not have a well homogeny. Further study should be conducted to explore more detailed information.

In summary, the correlation between NAION and CI is possible. The high ratio with CI in NAION patients indicates that the ocular disorder and the cerebral disorder remind each other. Hypertension is a risk factor for NAION patients with CI.
